# Allele Polymorphism and Haplotype Diversity of HLA-A, -B and -DRB1 Loci in Sequence-Based Typing for Chinese Uyghur Ethnic Group

**DOI:** 10.1371/journal.pone.0013458

**Published:** 2010-11-04

**Authors:** Chun-mei Shen, Bo-feng Zhu, Ya-jun Deng, Shi-hui Ye, Jiang-wei Yan, Guang Yang, Hong-dan Wang, Hai-xia Qin, Qi-zhao Huang, Jing-Jing Zhang

**Affiliations:** 1 The Key Laboratory of Environment and Gene Related to Diseases, Xi'an Jiaotong University, Ministry of Education, Xi'an, Shaanxi, People's Republic of China; 2 Blood Center of Shaanxi Province, Xi'an, Shaanxi, People's Republic of China; 3 The Key Laboratory of Ministry of Health for Forensic Sciences, School of Medicine, Xi'an Jiaotong University, Xi'an, Shaanxi, People's Republic of China; 4 Department of Forensic Sciences, School of Medicine, Xi'an Jiaotong University, Xi'an, Shaanxi, People's Republic of China; 5 Center of Forensic Sciences, Beijing Genomics Institute, Beijing, People's Republic of China; 6 Beijing Institute of Genomics, Chinese Academy of Science, Beijing, People's Republic of China; 7 Vascular Biology Center, Medical College of Georgia, Augusta, Georgia, United States of America; 8 Institute of Molecular Medicine and Genetics, Medical College of Georgia, Augusta, Georgia, United States of America; 9 The Second Team of Students Brigade, Third Military Medical University, Chongqing, People's Republic of China; 10 Beijing Genomics Institute, Beijing, People's Republic of China; St. Petersburg Pasteur Institute, Russian Federation

## Abstract

**Background:**

Previous studies indicate that the frequency distributions of HLA alleles and haplotypes vary from one ethnic group to another or between the members of the same ethnic group living in different geographic areas. It is necessary and meaningful to study the high-resolution allelic and haplotypic distributions of HLA loci in different groups.

**Methodology/Principal Findings:**

High-resolution HLA typing for the Uyghur ethnic minority group using polymerase chain reaction-sequence-based-typing method was first reported. HLA-A, -B and -DRB1 allelic distributions were determined in 104 unrelated healthy Uyghur individuals and haplotypic frequencies and linkage disequilibrium parameters for HLA loci were estimated using the maximum-likelihood method. A total of 35 HLA-A, 51 HLA-B and 33 HLA-DRB1 alleles were identified at the four-digit level in the population. High frequency alleles were HLA-A*1101 (13.46%), A*0201 (12.50%), A*0301 (10.10%); HLA-B*5101(8.17%), B*3501(6.73%), B*5001 (6.25%); HLA-DRB1*0701 (16.35%), DRB1*1501 (8.65%) and DRB1*0301 (7.69%). The two-locus haplotypes at the highest frequency were HLA-A*3001-B*1302 (2.88%), A*2402-B*5101 (2.86%); HLA-B*5001-DRB1*0701 (4.14%) and B*0702-DRB1*1501 (3.37%). The three-locus haplotype at the highest frequency was HLA-A*3001-B*1302-DRB1*0701(2.40%). Significantly high linkage disequilibrium was observed in six two-locus haplotypes, with their corresponding relative linkage disequilibrium parameters equal to 1. Neighbor-joining phylogenetic tree between the Uyghur group and other previously reported populations was constructed on the basis of standard genetic distances among the populations calculated using the four-digit sequence-level allelic frequencies at HLA-A, HLA-B and HLA-DRB1 loci. The phylogenetic analyses reveal that the Uyghur group belongs to the northwestern Chinese populations and is most closely related to the Xibe group, and then to Kirgiz, Hui, Mongolian and Northern Han.

**Conclusions/Significance:**

The present findings could be useful to elucidate the genetic background of the population and to provide valuable data for HLA matching in clinical bone marrow transplantation, HLA-linked disease-association studies, population genetics, human identification and paternity tests in forensic sciences.

## Introduction

Almost all the Uyghurs in China live in Xinjiang Uyghur Autonomous Region. The region, by far the biggest of the country's regions and provinces, covers more than 1,709,400 square kilometers or approximately one sixth of China's total landmass. Although Han, Kazak, Hui, Mongolian, Kirgiz, Tajik, Xibe, Ozbek, Manchu, Daur, Tatar and Russian people also live in Xinjiang, the Uyghur, who believe in Islam, are the largest ethnic group there (http://www.fmprc.gov.cn/eng/ljzg/3584/t17921.htm). The Uyghur ethnic minority has its own language and alphabet. The Uyghur language, formerly known as Eastern Turki, belongs to the Uyghur Turkic branch of the Turkic language family, which is controversially a branch of the Altaic language family. The Uyghurs have two written languages, with one based on Arabian letters and the other on Latin letters [1], [2].

The human major histocompatibility complex (MHC), also called human leukocyte antigen (HLA), is located at chromosome 6p21.31 [3]. HLA is the most gene-dense region and plays an important role in the generation of immune responses. According to the IMGT (the international ImMunoGeneTics information project)/HLA database (http://www.ebi.ac.uk/imgt/hla/stats.html, June 18, 2010), a total of 3391 alleles, including 1001 HLA-A, 1605 HLA-B and 785 HLA-DRB1 alleles, have been identified at HLA class I and class II loci in the world, which indicates that the HLA system constitutes the most complex and highly polymorphic genetic system in the human genome that has ever been discovered. The previously published population data [4]–[11] have revealed that the frequency distributions of HLA alleles and haplotypes vary from one ethnic group to another or between the members of the same ethnic group living in different geographic areas. Furthermore, each ethnic group is characterized by a unique pattern of high diversity genetic linkage disequilibria (LD) among HLA loci [6]. Therefore, the extensive allelic polymorphisms and the linkage disequilibrium among different HLA loci in different populations are usually used as highly polymorphic genetic markers in anthropology studies. Genetic distance calculation, cluster analysis and principal component analysis on the basis of the allelic frequencies at HLA loci in different populations have become valuable tools to study the genetic relationships among different ethnic groups as well as the origin, evolution and migration of the populations [12], [13].

Studies have been done on the genetic polymorphisms of HLA loci in the Uyghur population using low-middle resolution techniques. Yan CX et al. analyzed the HLA-A locus in Chinese Uyghur population by PCR amplification using sequence-specific oligonucleotide probe (PCR-SSOP) [14]; Mizuki N et al. studied the HLA class II (DRB1, DQA1, DQB1 and DPB1) genotyping in a Uyghur population in the Silk Route of Northwest China using the polymerase chain reaction-restriction fragment length polymorphism (PCR-RFLP) method [15]; Xu MY et al. investigated the low-resolution HLA-B locus polymorphisms in Xinjiang Uyghur ethnic group using the polymerase chain reaction-sequence specific primers (PCR-SSP) method [16]. However, those researchers studied the genetic polymorphisms of either the HLA class I or the class II in the Uyghur population, which means that their studies provided limited genetic information of the population. No high-resolution data using HLA class I and II loci of the Uyghur ethnic group in the same region are available now. In the present study, we investigated the allelic distributions at HLA-A, -B and -DRB1 loci, calculated the linkage disequilibrium parameters of two- or three-locus haplotypes, estimated the Haplotypic frequencies of HLA-A, -B and -DRB1 loci, and constructed a phylogenetic tree based on the allelic frequencies of HLA loci of the Uyghur ethnic minority group in Yining city, Xinjiang Uyghur Autonomous Region of China, using the polymerase chain reaction sequence-based typing (PCR-SBT) approach. The study is expected to enrich the knowledge of HLA genetic polymorphisms in Chinese ethnic groups and to further the understanding of the genetic relationships between Uyghur and other ethnic groups.

## Results

### Hardy-Weinberg Equilibrium tests of HLA-A, -B and -DRB1 loci

Polymorphisms of HLA-A, -B and -DRB1 loci in the Uyghur ethnic minority of Chinese Xinjiang Uyghur Autonomous Region were investigated using the SBT method. The *P* values for Hardy-Weinberg equilibrium tests of HLA-A, -B and -DRB1 loci were 0.5785, 0.9696 and 0.4242, respectively, which indicate that the HLA genotypic frequency distributions in the Uyghur ethnic minority were consistent with the Hardy-Weinberg equilibrium at the three HLA loci.

### Allelic frequency distributions at HLA-A, -B and -DRB1 loci

The allelic frequencies at HLA-A, -B and -DRB1 loci obtained by high-resolution DNA typing of 104 unrelated healthy Uyghur individuals are summarized in Table 1. Thirty-five distinct alleles were detected at HLA-A locus in this population. The allelic frequencies of HLA-A*2, A*11, A*24 and A*33 groups accounted for 17.78%, 16.34%, 11.05% and 9.62% of the total, respectively. The HLA-A*2 group was found to be the most diverse allelic family at HLA-A locus and consisted of nine alleles: A*0201, A*0202, A*0203, A*0205, A*0206, A*0211, A*0214, A*0222 and A*0236. The most common subtype HLA-A*1101 allele was seen at a frequency of 13.46%; the allelic frequencies of the next four most common alleles, A*0201, A*0301, A*3301 and A*2402, were 12.50%, 10.10%, 9.62% and 9.13%, respectively. The five most common alleles have a cumulative frequency of 54.81%.

**Table 1:** Allelic frequencies at HLA-A, -B and -DRB1 loci in the Uyghur ethnic minority in Xinjiang Uyghur Autonomous Region, China.

HLA-A	Allelicfrequencies	HLA-B	Allelicfrequencies	HLA-B	Allelicfrequencies	HLA-DRB1	Allelicfrequencies
0101	7.69%	0702	4.33%	4501	0.48%	0101	4.81%
0201	12.50%	0705	0.48%	4601	0.96%	0102	2.88%
0202	0.48%	0801	3.37%	4801	2.41%	0301	7.69%
0203	0.48%	0803	0.48%	4901	2.41%	0305	2.40%
0205	1.92%	0805	0.48%	5001	6.25%	0306	0.48%
0206	0.48%	1301	1.92%	5004	0.48%	0317	0.96%
0211	0.48%	1302	3.85%	5101	8.17%	0401	5.29%
0214	0.48%	1402	2.88%	5102	0.48%	0402	0.48%
0222	0.48%	1501	1.92%	5106	0.96%	0403	1.92%
0236	0.48%	1502	0.96%	5201	3.37%	0404	2.40%
0301	10.10%	1503	0.96%	5204	0.48%	0405	1.92%
0302	0.96%	1505	0.48%	5301	0.48%	0701	16.35%
0306	0.48%	1508	0.48%	5401	1.92%	0801	0.96%
1101	13.46%	1517	0.96%	5601	0.48%	0803	0.48%
1106	0.48%	1518	0.96%	5701	1.92%	0901	1.92%
1110	1.44%	1529	1.44%	5801	4.81%	1001	2.40%
1119	0.96%	1801	4.81%			1101	3.85%
2301	2.88%	2703	0.96%			1104	3.85%
2402	9.13%	2704	0.48%			1106	0.48%
2403	1.44%	2707	0.96%			1111	0.48%
2404	0.48%	3501	6.73%			1201	1.92%
2501	0.96%	3502	0.96%			1202	2.88%
2601	5.29%	3503	2.88%			1301	5.77%
2602	0.48%	3508	1.44%			1302	4.81%
2613	0.96%	3520	0.48%			1303	1.92%
2901	0.48%	3701	3.37%			1305	0.96%
3001	3.85%	3801	3.37%			1401	1.92%
3002	0.48%	3901	2.41%			1403	0.48%
3101	1.44%	4001	0.96%			1404	0.96%
3201	4.33%	4006	0.96%			1405	1.44%
3301	9.62%	4009	0.48%			1407	0.96%
3604	1.92%	4101	0.96%			1501	8.65%
6601	0.48%	4102	1.92%			1502	5.29%
6801	1.92%	4402	2.41%				
7401	0.48%	4403	1.44%				

HLA-B locus was extremely diverse, with a total of 51 alleles detected. The allelic frequencies of the HLA-B*35, B*51, B*15 and B*50 groups accounted for 12.49%, 9.61%, 8.16% and 6.73%, respectively. HLA-B*5101 had the highest frequency (8.17%) at the HLA-B locus, followed by B*3501 (6.73%), B*5001 (6.25%), B*1801 (4.81%), B*5801 (4.81%) and B*0702 (4.33%). The HLA-B*15 group was found to be the most diverse allelic family at HLA-B locus, consisting of eight alleles: B*1501, B *1502, B *1503, B *1505, B *1508, B *1517, B *1518 and B *1529.

Thirty-three HLA-DRB1 alleles were identified. The allelic frequencies of the HLA-DRB1*7, DRB1*15, DRB1*13, DRB1*4 and DRB1*3 groups accounted for 16.35%, 13.94%, 13.46%, 12.01% and 11.53%, respectively. The six most common HLA-DRB1 alleles with a frequency higher than 5% were DRB1*0701 (16.35%), DRB1*1501 (8.65%), DRB1*0301 (7.69%), DRB1*1301(5.77%), DRB1*0401(5.29%) and DRB1*1502 (5.29%). The cumulative frequency of the six alleles was 49.04%.

### HLA haplotypic frequencies and linkage disequilibriums

A total of 133 HLA A-B haplotypes, 118 HLA B-DRB1 haplotypes and 173 HLA A-B-DRB1 haplotypes were estimated using the expectation maximization (EM) algorithm. The estimated haplotypes were considered statistical significance only when their frequencies were greater than or equal to 2/3N (N: the sample size) [17]. Only 41 HLA A-B haplotypes, 37 HLA B-DRB1 haplotypes and 22 HLA A-B-DRB1 haplotypes had a haplotypic frequency higher than 0.64% and were thus considered statistically significant. The significant haplotypes of the three groups had a cumulative haplotypic frequency of 55.48%, 54.93% and 27.4%, respectively.

The haplotypic frequencies of the estimated significant HLA haplotypes (the haplotypic frequencies ≥1.00%) are summarized in Table 2. Three HLA-A-B haplotypes had a frequency greater than 2%, i.e., HLA-A*3001-B*1302 (2.88%), A*2402-B*5101 (2.86%) and A*3201-B*3501 (2.40%), followed by A*0101-B*3701 (1.92%), A*1101-B*3503 (1.92%), A*1101-B*0702 (1.92%), A*0205-B*5001(1.92%), A*2301-B*4901(1.92%), A*2601-B*3801 (1.92%) and A*3301-B*1402 (1.92%). Seven HLA-B-DRB1 haplotypes showed a frequency greater than 2%, i.e., HLA-B*5001-DRB1*0701 (4.14%), B*0702-DRB1*1501 (3.37%), B*3501-DRB1*0701 (2.88%), B*1302-DRB1*0701 (2.83%), B*1402-DRB1*0102(2.40%), B*5201-DRB1*1502 (2.40%) and B*0801-DRB1*0301 (2.40%), followed by B*3701-DRB1*1001(1.92%) and B*5801-DRB1*0301(1.92%). The most common HLA-A-B-DRB1 haplotypes with a frequency higher than 1.9% were HLA-A*3001-B*1302-DRB1*0701(2.40%), HLA-A*1101-B*0702-DRB1*1501(1.92%), HLA-A*0205-B*5001-DRB1*0701(1.92%), HLA-A*3201-B*3501-DRB1*0701(1.92%) and HLA-A*3301-B*1402-DRB1*0102(1.92%).

**Table 2:** Estimated main high-frequency HLA haplotypes and their frequencies of the Uyghur ethnic minority in Xinjiang Uygur Autonomous Region, China*.

HLAA-B-DRB1	Haplotypic frequencies	HLAA-B	Haplotypic frequencies	HLAB-DRB1	Haplotypic frequencies
3001-1302-0701	2.40%	3001-1302	2.88%	5001-0701	4.14%
1101-0702-1501	1.92%	2402-5101	2.86%	0702-1501	3.37%
0205-5001-0701	1.92%	3201-3501	2.40%	3501-0701	2.88%
3201-3501-0701	1.92%	0101-3701	1.92%	1302-0701	2.83%
3301-1402-0102	1.92%	1101-3503	1.92%	1402-0102	2.40%
1101-3503-1301	1.44%	1101-0702	1.92%	5201-1502	2.40%
0301-5801-0301	1.44%	0205-5001	1.92%	0801-0301	2.40%
		2301-4901	1.92%	3701-1001	1.92%
		2601-3801	1.92%	5801-0301	1.92%
		3301-1402	1.92%	1801-1101	1.44%
		0301-3501	1.68%	1801-1104	1.44%
		0301-5801	1.68%	3503-1301	1.44%
		1101-5201	1.44%	4102-1303	1.44%
		0201-1801	1.44%	4801-1301	1.44%
		2402-3901	1.44%	5101-0401	1.44%
		0301-5001	1.44%	5101-0404	1.44%
		3301-5801	1.44%	5101-0301	1.38%
		2601-0801	1.41%	5801-0701	1.15%
		0201-5101	1.33%	5101-0701	1.02%

*Listed are only the HLA haplotypes with the haplotypic frequency≥1.00%.

Table 3 shows the values of haplotypic frequency (HF), absolute linkage disequilibrium (ALD), maximal linkage disequilibrium (MLD) and relative linkage disequilibrium parameter (RLD) in the population, which have been commonly used in the evaluation of the linkage disequilibrium (LD) in population genetics. HLA-A*0201, A*0205 and A*2501 alleles were found to be tightly associated with HLA-B*4006, B*5001 and B*1801, respectively, with their relative linkage disequilibrium values equal to 1. The HLA-A*2301, A*3001 and A*3604 alleles were also found to have association with HLA-B*4901, B*1302 and B*1529, with their corresponding relative linkage disequilibrium values equal to 0.7920, 0.7400 and 0.6612, respectively. HLA-A*3301 showed a strong linkage disequilibrium with HLA-B*1402 and B*4403. HLA-B*2703, B*5106 and B*1517 had significant linkage disequilibrium with DRB1*0401, DRB1*1502 and DRB1*1302, respectively, with the relative linkage disequilibrium parameter equal to 1. The relative linkage disequilibrium values of HLA-B*1402-DRB1*0102, HLA-B*3701-DRB1*1001, HLA-B*0702-DRB1*1501 and HLA-B*4102-DRB1*1303 were higher than 0.70%, which means HLA-B*1402, B*3701, B*0702 and B*4102 had tight associations with HLA-DRB1*0102, DRB1*1001, DRB1*1501 and DRB1*1303, respectively.

**Table 3:** Haplotypic frequencies and linkage disequilibrium parameters of HLA two-locus haplotypes in the Uyghur ethnic minority in Xinjiang Uyghur Autonomous Region, China.

HLA A-B	HF	ALD	MLD	RLD	HLA B-DRB1	HF	ALD	MLD	RLD
0201-4006	0.96%	0.84%	0.84%	1.0000	2703-0401	0.96%	0.91%	0.91%	1.0000
0205-5001	1.92%	1.80%	1.80%	1.0000	5106-1502	0.96%	0.91%	0.91%	1.0000
2501-1801	0.96%	0.92%	0.92%	1.0000	1517-1302	0.96%	0.91%	0.91%	1.0000
2301-4901	1.92%	1.85%	2.34%	0.7920	1402-0102	2.40%	2.32%	2.80%	0.8297
3001-1302	2.88%	2.74%	3.70%	0.7400	3701-1001	1.92%	1.84%	2.32%	0.7930
3604-1529	0.96%	0.93%	1.41%	0.6612	0702-1501	3.37%	2.99%	3.96%	0.7561
3301-1402	1.92%	1.65%	2.60%	0.6324	4102-1303	1.44%	1.41%	1.88%	0.7463
3301-4403	0.96%	0.82%	1.30%	0.6324	5201-1502	2.40%	2.23%	3.19%	0.6973
1101-3503	1.92%	1.54%	2.49%	0.6161	0801-0301	2.40%	2.14%	3.11%	0.6894
2402-3901	1.44%	1.22%	2.19%	0.5581	1302-0701	2.83%	2.20%	3.22%	0.6818
2601-3801	1.92%	1.74%	3.19%	0.5467	1529-0403	0.96%	0.93%	1.41%	0.6612
0101-3701	1.92%	1.66%	3.11%	0.5349	4403-0701	0.96%	0.73%	1.20%	0.6028
3201-3501	2.40%	2.11%	4.04%	0.5235	5001-0701	4.14%	3.12%	5.23%	0.5971
					4801-1301	1.44%	1.30%	2.27%	0.5739
					5101-0404	1.44%	1.25%	2.21%	0.5644

HF: haplotypic frequency; ALD: absolute linkage disequilibriums; MLD: maximal linkage disequilibriums; RLD: relative linkage disequilibrium parameters.

### Construction of a phylogenetic tree

**Figure 1:**
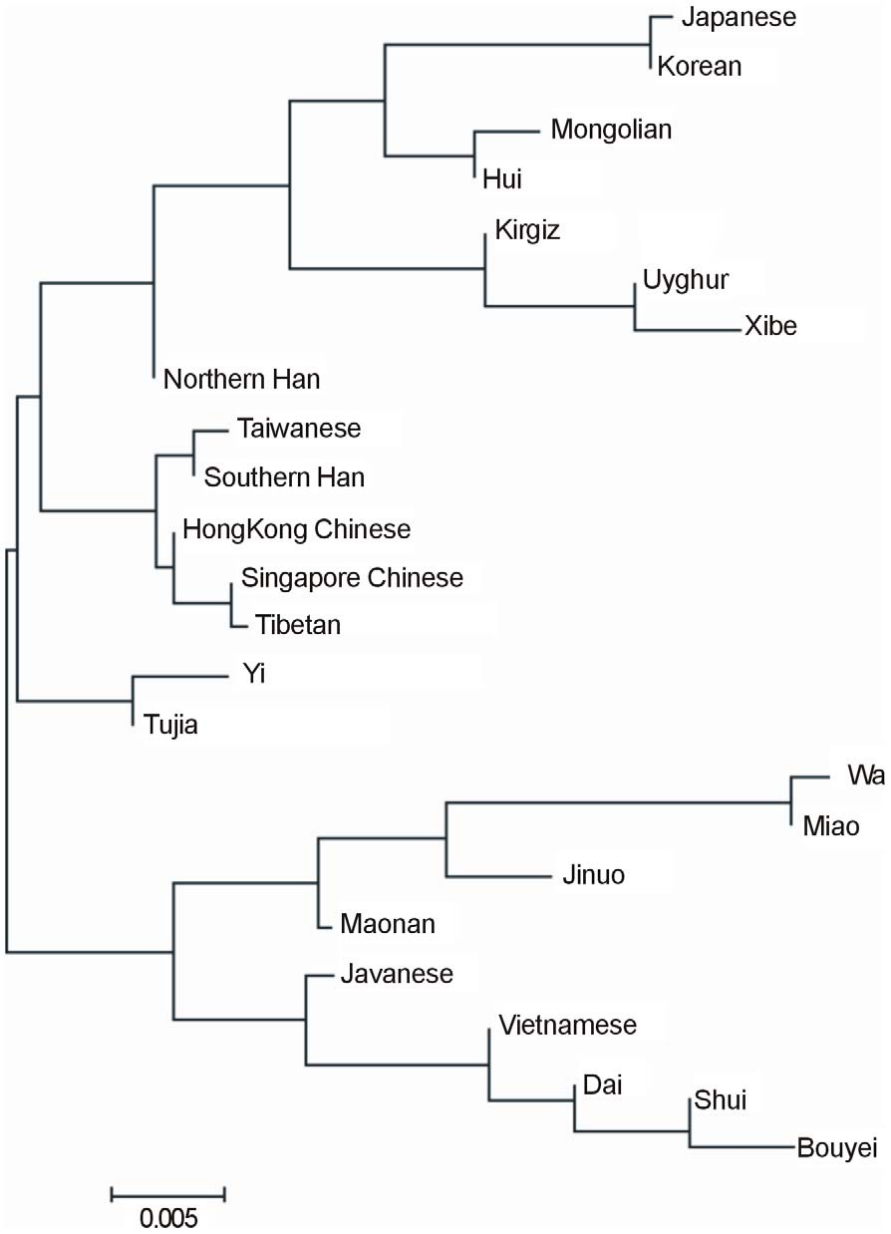
Dendrogram constructed by the neighbor-joining method shows the relationships of the Uyghur population with other 23 populations based on the allelic frequencies of HLA-B locus.

The phylogenetic tree in Figure 1 was constructed using the allelic frequencies at HLA-B locus in the Xinjiang Uyghur population and its neighboring ethnic groups. The neighboring ethnic groups used in the phylogenetic tree construction were Miao, Bouyei and Shui ethnic minorities [6], Jinuo and Wa populations [18], Maonan people [19], Yi ethnic minority [13], Tujia nationality [20], Dai population [21], Tibetan ethnic population [22], Mongolian and Hui ethnic groups [23], Kirgiz and Xibe ethnic minority (http://202.117.24.55:8001/xwlw/detail_xwlw.jsp?searchword_AUTHOR%3D%C9%F2%B4%BA%C3%B7&singlesearch_no&channelid_65004&record_2), Hong Kong Chinese population[24], Han from Southern China[25], Han population in Northern China [26], Taiwanese [27], Singapore Chinese [28], Javanese [29], Vietnamese population(the Kinh population in Vietnam)[30], Korean population [31] and Japanese population [32]. The three main clusters of populations obtained are: (1) the populations living in Korea, Japan and the northwestern of China, (2) the Han populations in different regions, and (3) the populations living in Vietnam, Indonesia and the southeastern of China. The Uyghur population was first clustered with Xibe group, and then with Kirgiz, Hui, Mongolian and other groups. According to the phylogenetic tree, the Uyghur population showed the closest genetic structure to the northwestern Chinese populations.

### Forensic parameters

The values of the major forensic parameters for HLA-A, -B, and -DRB1 loci in the Uyghur population were listed in Table 4. The forensic parameters showed high values, with heterozygosity observed (HO) and expected heterozygosity (HE) equal to 0.9231 and 0.9135 for HLA-A locus, 0.9808 and 0.9681 for HLA-B locus, and 0.9519 and 0.9231 for HLA-DRB1 locus, respectively. All HLA loci were highly polymorphic (polymorphism information contents, PIC >0.5). HLA-A locus was the relatively low polymorphic of the three HLA loci (PIC  = 0.9181) and HLA-B locus was the most polymorphic (PIC  = 0.9624). The power of discrimination (PD) ranged from 0.9784 (HLA-A locus) to 0.9887 (HLA-B locus), whereas the probability of paternity exclusion (PPE) ranged from 0.8230 (HLA-A locus) to 0.9353 (HLA-B locus).

**Table 4:** Forensic parameters for HLA-A, -B and -DRB1 loci in the Uyghur ethnic minority group.

Locus	HO	HE	PD	PIC	PPE	*p*
**HLA-A**	0.9231	0.9135	0.9784	0.9181	0.8230	0.5785
**HLA-B**	0.9808	0.9681	0.9887	0.9624	0.9353	0.9696
**HLA-DRB1**	0.9519	0.9231	0.9784	0.9338	0.8428	0.4242

HO: observed heterozygosity; HE: expected heterozygosity; PD: power of discrimination; PIC: polymorphism information contents; PPE: probability of paternity exclusion. *P*: probability values of exact tests for Hardy-Weinberg equilibrium.

## Discussion

With the continuous development of economy and society and the progress in human culture, inter-ethnic intermarriages, especially the marriage between national minorities and the Han population have markedly increased, resulting in the assimilation and the decrease in ethnic minority populations. In addition, the ethnic minority populations previously isolated in remote areas are gradually migrating to the civilized areas in pursuit of a better life. These people may also marry other ethnic people. In view of these facts, the number of pure blooded ethnic minorities decrease rapidly. Therefore, preservation and study of genetic information resources of pure blooded ethnic minorities have been a priority on the research agenda. The authors have collected and stored blood samples from more than ten national minorities and have studied the population genetics of HLA loci and the short tandem repeats (STR) of Y chromosome and autosome in these national minorities, including the genetic polymorphisms of Y chromosome and autosome STR in the Uyghur ethnic minority in Xinjiang Uyghur Autonomous Region, China [33], [34]. The authors have also studied the HLA polymorphisms (low resolution) of the Mongol ethnic group in Inner Mongolia [35], the Hui population in Ningxia Hui autonomous region [36], and the Han population in Guanzhong region of the Shaanxi province [12] by using the SSO and SSP methods, and the HLA polymorphisms (high resolution) of Yi population in Yunnan [13] and Han population in Beijing by using the SBT method [37]. In this study, the distributions of HLA class I and class II allelic and haplotypic frequencies in 104 unrelated Uyghur individuals living in Yining city of the Xinjiang Uyghur Autonomous Region, China were analyzed for the first time by the high-resolution PCR-SBT method.

A total of 35 HLA-A, 51 HLA-B and 33 HLA-DRB1 alleles defined at the four-digit level were identified in the population. HLA-A*1101 was the most frequent allele at HLA-A locus in the Uyghur population, followed by A*0201, A*0301, A*3301 and A*2402. The five alleles accounted for 54.81% of the total HLA-A allelic frequency. HLA-A*1101 is also the most common HLA-A allele in other populations such as Chinese Wa (58.4%) [18], Dai (39.1%) [21], Jinuo (36.7%) [18], Miao (35.9%) [6], Maonan (35.2%) [19], Bouyi (31.4%) [6], Shui (29.5%) [6], Yi (26.3%) [13], Drung (19.8%) [38], Uygur (19.7%) [14], Mongolian (16.2%) [23], Hui (15.9%) [23], Tujia (14.7%) [20], Tibetan (13.0%) [22], isolated Han population in Southwest China (31.7%) [39], Meizhou Han (30.3%) [40], Hong Kong Chinese population (28.7%) [24], Taiwanese (27.5%) [27], Han from Southern China (26.7%) [25], Beijing Han (20.23%) [37], Kinh population in Vietnam (22.9%) [30], Sundanese–Javanese (Indonesia) (16.42%) [29], Javanese (13.89%) [29], Korean (10.8%) [31], Xibe (8.54%), Japanese (8.2%) [32], Kirgiz (6.99%), the population on Madeira Island (Portugal) (5.9%) [41], German (5.06%) [42], Eastern European Americans (6.3%) [43] and Mexican Americans (4.2%) [44]. The HLA-A*1101 allelic frequency of Han population in the south of China is higher than those of Han population in the north of China, Japanese and Korean. The HLA-A*1101 allelic frequency distributions of the national minorities in the southwest of China are relatively higher than those of the national minorities in the northwest of China. HLA-A*0201 allele, which was the second most frequent allele in the Uyghur population, has also been detected in the Han populations living in the different regions and in some ethnic minorities in the northwest of China such as Kirgiz (18.3%), Hui (17.3%), Tibetan (21.8%), Mongolian (12.7%), Xibe (7.93%), and in Euro-American populations (with a high frequency distribution of more than 20%). However, HLA-A*0201 allele is less common in some ethnic minorities in the southwest of China. The high frequencies of HLA-A*0301 and A*3301 alleles in the Uyghur population are rarely or never seen in other populations. The fifth high frequent allele in the Uyghur population was A*2402. The HLA-A*2402 allelic frequency ranges from 8.1% to 19.6% in most Han populations from different regions, some ethnic minorities in the northwest and southwest of China, and the populations of Euro-American countries. The HLA-A*2402 allele shows higher frequency distributions in Koreans (21.65%), Japanese (37.9%), Meizhou Han (22.2%), Shui (24.3%), Tibetan (27.2%) and Yi population(32.4%).

The HLA-B locus exhibited the highest degree of polymorphisms in the Uyghur population. The three alleles with a frequency of more than 5% detected at the HLA-B locus were HLA-B*5101, B*3501 and B*5001. The HLA-B*5101 is also the most frequent allele in Asians, such as Kirgiz, Xibe, Tibetan, Dai, Mongolian, Hui, isolated Han population in southwest China, Beijing Han, Korean and Japanese. The HLA-B*3501 and B*5001 alleles detected in the Uyghur population are less common or never seen in other Asian populations. The HLA-B*4001, B*4601, B*1501 and B*1502 alleles, which have been found to have high frequencies in the Asian populations previously reported [6], [13], [18], [25], had a low frequency of less than 2% in the Uyghur population. The Uyghur population showed a great variability in the allelic frequency distributions at HLA-B locus, resulting in the difficulty in briefly describing the distribution characteristics of HLA-B allelic frequencies in the Uyghur population.

The HLA-DRB1 locus exhibited a high degree of polymorphism in the Uyghur population. The HLA-DRB1*0701, DRB1*1501, DRB1*0301, DRB1*1301, DRB1*0401 and DRB1*1502 were predominant alleles in the HLA-DRB1 locus in the population. The results were similar to frequency distributions of DRB1*0701 (16.7%) and DRB1*0301 (14.0%) in the Uygur population in the Silk Route of Northwest China [15] and DRB1*0301 (13.1%) and DRB1*0701(10.7%) in the Kazak population in the Silk Route of Northwest China [45]. These HLA data indicate that the Uyghur ethnic group shows a relatively close genetic relationship to the Kazak population inhabiting the same area. The HLA-DRB1*0701 was found to be the most frequent allele in Uyghur ethnic group, as well as in some other populations, such as Han population in Beijing, Southern Han, Korean population, the population on Madeira Island (Portugal), Caucasian population on Northern Ireland [46]. However, it is rare or less common in Dai, Jinuo, Wa, Maonan, Drung, Naxi, Miao and Yao ethnic groups in the southwest of China. HLA-DRB1*1501 and DRB1*0901, two high frequency alleles in the Chinese population, exist in most ethnic groups in the north and south of China, with an average allelic frequency of more than 8.5%. The HLA-DRB1*1501 was the second most frequent allele in the Uyghur population whereas allelic frequency of DRB1*0901 was only 1.92% in the study population and had a very low value in the European and American populations.

Three HLA-A-B haplotypes, HLA-A*3001-B*1302, HLA-A*2402-B*5101 and HLA-A*3201- B*3501, occurred at a frequency greater than 2% in the Uyghur population. HLA-A*3001-B*1302 detected in the study population have commonly been observed in Kirgiz (2.72%), Hong Kong Chinese population (1.30%), Taiwanese (1.2%) and Xibe population (1.19%). HLA-A*2402-B*5101 in the Uyghur population has been found in Kirgiz (2.72%), Hong Kong Chinese population (1.30%) and Xibe population (1.19%). HLA-A*3201-B*3501 at a high frequency in the population is less frequent in other populations. HLA-A*1101-B*0702 and HLA-A*1101-B*5201 at a high frequency detected in the study population have been observed in the Yi population, at a respective frequency of 1.32% and 4.37%. HLA-A*0301-B*3501, HLA-A*0201-B*1801 at a high frequency in the population have been seen at a frequency of 2.01% and 2.05% in Eastern European American, respectively. HLA-B*5801-DRB1*0301 at a high frequency in studied population is also the most frequent two-locus haplotypes in other populations such as the Kinh population in Vietnam (4.10%), Han from Southern China (3.00%), Dai (4.40%) and Maonan (4.20%). The Uyghur population shared some haplotypes with other populations, namely, HLA-B*1302-DRB1*0701 (2.83%) with Korean population (2.89%) and Taiwanese (1.27%); HLA-B*3701-DRB1*1001 (1.92%) with Korean population (1.34%), Han from Southern China (3.00%); and HLA-B*5201-DRB1*1502 (2.40%) with Korean population (2.37%). HLA-A*3001-B*1302-DRB1*0701 (2.40%) in the population was also present at a high frequency in both Korean population (2.68%) and Taiwanese (3.02%). In all, the distributions of these haplotypes further suggest that the Uyghur population was a characteristic northwestern Chinese population. The haplotypes in this study were estimated using EM algorithm, but the haplotypes presented were not based on family data; therefore, we need to acknowledge the potential errors inherent to haplotype estimation methods [47].

The phylogenetic tree reveals that the Uyghur population belonged to the northwestern Chinese populations and was most closely related to Xibe group, and then to Kirgiz, Hui, Mongolian, and Northern Han. The reason that HLA-B locus was chosen for the phylogenetic tree construction was that HLA-B locus is highly polymorphic and that high-resolution HLA-B data can be obtained from a large number of comparable populations. The similar clustering results were obtained using the single HLA-A or HLA-DRB1 locus or HLA A-B-DRB1 haplotype. The close relationship of the Uyghur population with these groups may be partly explained by its history. The origin of the Uyghur population can be traced back to the Dingling nomads in the third century BC. The majority of the Uyghurs moved to the Western Region (present-day Xinjiang area) and some went to the Tufan principality in western Gansu Province after the mid-ninth century. The Uyghurs who settled in the Western Region intermarried with the Han people in Southern Xinjiang and Tibetan, Qidan and Mongol tribes, and evolved into the group now known as the Uyghurs.

Genetic distance here refers to the genetic divergence between populations within a species. A small genetic distance indicates a close genetic relationship between two populations whereas a large genetic distance indicates a distant genetic relationship [48]. The results of the genetic distances between the Uyghur population and other populations agreed with clustering analytic results of the Uyghurs and these populations. The Uyghur had the lowest genetic distance with the Kirgiz ethnicity (0.0089), followed by Mongolian (0.0112), Hui (0.01201), Xibe (0.01204), Northern Han (0.0165), Korean (0.0178), Japanese (0.0248), Tibetan (0.0265), Southern Han (0.0325), Tujia (0.0346), Vietnamese (0.0356), Yi (0.0360), Javanese (0.0377), Singapore Chinese (0.0385), HongKong Chinese (0.0417), Taiwanese (0.0454), Wa (0.0467), Dai (0.0551), Bouyei (0.0581), Maonan (0.0619), Miao (0.0630), Shui (0.0673) and Jinuo (0.0711). The results show that the Uyghur population had a close genetic relationship with Kirgiz, Mongolian, Hui, Xibe and Northern Han, but a relatively distant genetic relationship with Dai, Bouyei, Maonan, Miao, Shui and Jinuo in Southwestern China. The best explanation for the close genetic relationship of the Uyghur population with Kirgiz and Xibe ethnic groups is that these populations live in the same region and that gene flows occur at a significantly high level among the populations. These results agree with those of some previous studies. Genetic landscape through 14 single nucleotide polymorphisms (SNP) and 12 Y-STR loci shows that the Uyghur is closer to the Han Chinese and Mongolian population [49]. The dendrogram based on the allelic frequencies of the four variable number of tandem repeats (VNTR) and one STR loci reveals that Uyghur, Hui, Northern Han and Japanese form one cluster [50]. Yu MS et al. analyzed Mitochondrial DNA polymorphism using RFLP and found that the Uyghur was relatively close to the Han and the Hui populations [51]. The genetic relationships between Uyghur and Xibe [52], [53], Mongolian [53], [54], Hui [53], [55] and Tibetan [56] were also supported by Y-chromosome STR variation analysis.

The polymorphisms of genetic markers can be evaluated by some forensic parameters such as HO, HE, PD, PIC and PPE. A higher heterozygosity means that more allele diversity exists and therefore, there is less chance of a random sample matching. A locus is considered highly polymorphic when its PIC is higher than 0.5 [57]. PD and PPE are indicators for discrimination capability of a genetic marker and the PD and PPE values of a marker with high polymorphism are normally higher than 0.8 and 0.5, respectively [13]. Forensic parameters HO, HE, PD, PIC and PPE in the Uyghur population were higher than 0.82. The combined probability of exclusion, power of discrimination, probability of matching value for HLA-A, -B and -DRB1 loci were 0.998199, 0.999994 and 5.27×10^−6^, respectively, which suggest that allelic frequencies at HLA loci were highly polymorphic in the study population and that HLA loci could be applied to personal identification and paternity testing in forensic science. Take a paternity testing case in forensic science for example. When one or two STR loci violate the genetic principle in paternity testing and exclude the alleged father or mother as biological father or mother, it is suspected that the alleged father or mother may not be the biological father or mother. However, we considered that gene mutation might occur at the STR loci excluded. In such a case, additional genetic markers are needed to further determine paternity. Nowadays, although a large number of new STR loci have been studied, they don't adopt quality and measurement attestation in the forensic application. Therefore, the highly polymorphic HLA loci may be the best option for further paternity testing. The results show that combinations of the HLA loci and STR loci may be a powerful tool for individual identification and paternity testing for the Chinese Uyghur population in the region.

This present study may provide basic and valuable data for anthropological analysis and studies of HLA-associated disease susceptibility, organ transplantation (especially bone marrow transplantation), population genetics, human identification and paternity testing in forensic sciences.

## Materials and Methods

### Ethical statement

This study was approved by the Ethics Committee of Xi'an Jiaotong University, China. All the participants provided their written informed consent for the collection of the samples and the subsequent analysis, and the investigation was conducted in accordance with humane and ethical research principles of Xi'an Jiaotong Univeristy, China.

### Population samples

One hundred and four unrelated healthy Uyghur individuals were randomly chosen from Yining city, Xinjiang Uyghur Autonomous Region, China. All participants were interviewed to ensure that no individuals have common ancestry going back at least three generations.

### DNA extraction

Whole blood samples were collected from the participants and stored at -20°C until DNA extraction. Genomic DNA was extracted from whole blood containing ethylenediaminetetraacetic acid (EDTA) using a standard salting out method which yielded good quality high molecular weight DNA suitable for sequencing [58].

### Polymerase chain reaction-sequence based typing of HLA-A, -B and -DRB1 loci

All individuals were typed for HLA-A, -B and HLA-DRB1 loci. Sequencing-based-typing (SBT) of exons 2 and 3 at HLA-A and -B loci were performed according to Kurz et al. [59] and Pozzi et al. [60], with minor modifications; SBT of exon 2 and part of intron sequences on both sides of exon 2 at HLA-DRB1 locus were performed as described by Jia et al. [61] and Deng et al. [62].

PCR amplification was performed using a GeneAmp PCR system 9700 (Applied Biosystems, Foster City, CA, USA). PCR-amplified DNA fragments were purified and sequenced with ABI PRISM BigDye Terminator Cycle Sequencing Ready Reaction Kits (Applied Biosystems, Foster City, CA, USA) using an ABI 3730XL DNA sequencer (Applied Biosystems, Foster City, CA, USA), according to the manufacturer's instructions. Sequencing was processed always in the forward and reverse directions using software Sequencing Analysis, MatchTools and Navigator (MatchTools Allele Identification package, Applied Biosystems, Foster City, CA, USA). The software was used to detect the heterozygous position within each electropherogram and to assess the typing, based on an alignment of the processed sequence with a library of HLA sequences and alleles updated to October 2007. Ambiguous types were resolved to four digits according to the updated database (IMGT release 2.19.0).

### Statistical analysis

Allelic frequencies of HLA-A, -B and -DRB1 loci were estimated using SPSS 11.0 software (SPSS Inc., Chicago, Illinois). Haplotypic frequencies were calculated from genotype data by expectation maximization (EM) algorithm using Arlequin software package version 3.0 (Laurent Excoffier, CMPG, Zoological Institute, University of Bern, Switzerland) [63]. The linkage disequilibrium, the non-random association of two alleles at two different loci which is defined as the delta (D') coefficient, was calculated as described by John Lee [64]. The genetic distances between different populations were calculated as previously described by Nei [65] and a phylogenetic tree was constructed based on the allelic frequencies of HLA-B locus with the Mega 3.1 Software package (Center for Evolutionary Functional Genomics, the Biodesign Institute Tempe, AZ, USA) using the Neighbor-joining method [66]. The exact testing method of Guo and Thomson [67] was used to evaluate the deviation from the expected Hardy-Weinberg genotypic proportions.

Forensic parameters, including heterozygosity observed, expected heterozygosity, power of discrimination, polymorphism information contents, and probability of paternity exclusion, computed using the PowerStat version 1.2 spreadsheet (Promega Corporation, USA), as described by Tereba [68], were used to evaluate the polymorphisms of genetic markers. HO was defined as the number of heterozygote divided by the sample size and HE is defined as the estimated fraction of all individuals who would be heterozygous for any randomly chosen locus. PIC indicates the polymorphism of a locus and is often used to measure the indicative strength of genetic markers for linkage studies. PD is defined as the probability that two randomly selected individual will have different genotypes. PPE is defined as the fraction of the individuals that is different from that of a randomly selected individual or as the power of a locus to exclude a person from the possibility of being the biological father [69].
